# Effect of different concentrations of specific inhibitor of matrix metalloproteinases on the shear bond strength of self-adhesive resin cements to dentin

**DOI:** 10.4317/jced.53486

**Published:** 2017-03-01

**Authors:** Mohammad-Esmaeel Ebrahimi-Chaharom, Mehdi Abed-Kahnamoui, Mahmoud Bahari, Hamed Hamishehkar, Mahya Gharouni

**Affiliations:** 1 Dental and Periodontal Research Center, Dental Faculty, Tabriz University of Medical Sciences, Tabriz, Iran; 2 Associate Professor, Department of Operative Dentistry, Dental Faculty, Tabriz University of Medical Sciences, Tabriz, Iran; 3 Assistant Professor, Department of Operative Dentistry, Dental Faculty, Tabriz University of Medical Sciences, Tabriz, Iran; 4 Associate Professor of Pharmaceutical Sciences, Pharmaceutical Technology Laboratory, Drug Applied Research Center, Medical Research AND Development Complex, Tabriz University of Medical Sciences, Tabriz, Iran; 5 General Practitioner, Department of Operative Dentistry, Dental Faculty, Tabriz University of Medical Sciences, Tabriz, Iran

## Abstract

**Background:**

Considering the probability of chemical and enzymatic reactions between matrix metalloproteinases (MMPs) in the dentin structure and their specific inhibitors, the aim of the present study was to evaluate the effect of different concentrations of specific inhibitor of MMPs (galardin) on the shear bond strength of self-adhesive resin cements to dentin.

**Material and Methods:**

Forty-eight sound human premolars were mounted in self-cured acrylic resin after removal of the enamel on the buccal and lingual surfaces. The dentin surfaces achieved were polished and prepared with 600-grit silicon carbide paper. The samples were divided into 3 groups (n=16) based on the concentration of galardin used (with no galardin, galardin at a high concentration and galardin at a low concentration). In addition, 96 composite resin blocks, measuring 3 mm in height and diameter, were prepared. The composite resin blocks were bonded to the buccal and lingual surface dentin with Rely-X Unicem (RXC) and Speed CEM (SPC) self-adhesive resin cements, respectively, according to manufacturers’ instructions. After 24 hours of storage in distilled water at 37°C, the shear bond strength values were determined in MPa and fracture modes were evaluated under a stereomicroscope. Data were analyzed with two-way ANOVA and post-hoc Bonferroni test (α=0.05).

**Results:**

The shear bond strength of galardin at high concentration was significantly higher than that in the control group and galardin at a low concentrations (*P*<0.001). In addition, galardin at a low concentration exhibited higher shear bond strength compared to the control group (*P*=0.005). Furthermore, higher shear bond strength values were reported with the use of RXC compared to SPC (*P*<0.001).

**Conclusions:**

Irrigation with galardin increased the shear bond strength of self-adhesive resin cements to dentin and this increase had a direct relationship with the concentration of galardin in the solution.

** Key words:**N-(2(R)-2-(hydroxamidocarbonylmethyl)-4-methylpentanoyl)-L-tryptophan methylamide, Matrix metalloproteinase inhibitors, Dentin, Extracellular matrix, Luting agents, Dental Bonding.

## Introduction

Hydrophobic solvents such as acetone and ethanol are incorporated into dentin adhesives in order to facilitate penetration of monomer into the etched dentin and for direct contact of the resin with collagen fibers. As a result of the use of these solvents, the resin penetrates into the porous collagen network and forms a layer after curing, which is referred to as the hybrid layer (1). Durability and integrity of this collagen matrix is necessary for the long-term preservation of the bond to dentin (2).

Matrix metalloproteinases (MMPs) that are naturally found within the dentin matrix are responsible for the hydrolysis of the collagen matrix of the hybrid layer over time. MMPs are a group of Zn- and calcium-dependent enzymes that can hydrolyze natural collagen structures at a neutral pH value (3). It is hypothesized that these proteases have an important role in hydrolysis of dentin collagen fibers of hybrid layer (4-6).

It has recently been shown that the collagenolytic and gelatinolytic activities (5) of human dentin matrix are inhibited by nonspecific proteinase inhibitors such as chlorhexidine (7). Chlorhexidine is effective in the preservation of the hybrid layer *in vivo* and *in vitro* (4,8). It is believed that the specific inhibitors of MMPs that implement their inhibitory activities normally at low concentrations have a stronger effect on increasing the longevity of the hybrid layer compared to Chlorhexidine (9).

Galardin (Ilomastat, GM6001) (10,11) is a specific inhibitor of MMPs, with inherent inhibitory effects on MMPs 1, 2, 3, 8 and 9. Its activity was reported for the first time by Grobelny in 1990 (12,13). Galardin is a collagen-like structure that is attached to the active portion of MMPs and the hydroxamate structure and chelates the Zn ion in the catalytic tail of MMPs (14).

Breschi *et al.* showed that use of 0.2-mmol aqueous solution of galardin as a dentin irrigant before the bonding procedure does not improve the immediate bond strength of etch-and-rinse adhesive systems; however, it prevents the degradation of the bond in the long term (9). However, Almahdy *et al.* showed that incorporation of specific inhibitors of MMPs, such as Batimastat and galardin, into the adhesive systems results in an increase in the immediate bond strength and sealing ability (15).

Resin cements are the materials of choice for the cementation of indirect restorations. The principal and common mechanism of bonding of resin cements to enamel and dentin involves the use of adhesive systems that improve the micromechanical bond to the etched enamel and form a hybrid layer on the dentin (2,16). This strategy has high technique sensitivity and consists of several steps that result in a decrease in bonding efficacy and hypersensitivity after restoration. However, in self-adhesive resin cements the adhesive and the cement have been incorporated into one step so that decalcification and penetration into the matrix occur simultaneously, eliminating the need for prior preparation of the tooth surface (17).

According to Di Hipólito *et al.* pre-treatment with chlorhexidine adversely affects immediate bond strength of self-adhesive resin cements to dentin (18). So, the aim of present study was to evaluate the effect of different concentrations of galardin on the shear bond strength of self-adhesive resin cements to dentin.

## Material and Methods

-Specimen Preparation

After the study protocol has been approved by ethics committee of medical sciences university of Tabriz, Iran; a total of 48 sound human premolar teeth with closed apices, extracted for orthodontic reasons, from subjects 15-25 years of age, were selected for the purpose of this *in vitro* study. The teeth were immersed in 0.5% chloramine T solution immediately after extraction and preserved for at most 3 months until used for the purpose of the study after being mounted in self-cured acrylic resin.

A diamond saw in a high-speed handpiece was used to expose the dentin surface by abrading the enamel on the facial and lingual surfaces of the teeth. All the samples were evaluated under a stereomicroscope (Nikon, SMZ800, Tokyo, Japan) to make sure of the absence of enamel. The buccal and lingual surfaces of the teeth were polished and prepared with wet 600-grit SiC paper for 60 seconds for a uniform surface roughness in all the samples. A total of 96 composite resin blocks (Valux Plus, 3M ESPE, St. Paul, MN, USA), measuring 3 mm in height and diameter, were also prepared for bonding to dentin surfaces with the use of self-adhesive cements.

Then, the samples were divided into 3 groups based on the concentration of galardin (Sigma, Dorset, UK). Furthermore, the samples in each group were divided into two subgroups (n=8) based on the type of the adhesive cement used: Rely-X Unicem (RXC) (3M ESPE, St. Paul, MN, USA) and Speed CEM (SPC) (Ivoclar Vivadent, Schaan, Liechtenstein, Germany). The particulars of the self-adhesive resin cements used in the present study are presented in  Table 1.

**Table 1:**
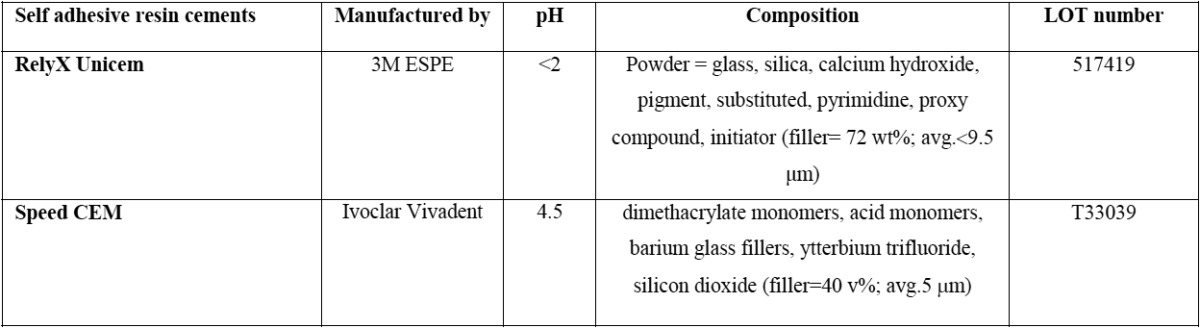
The particulars of the self-adhesive resin cements used in the present study.

•Control group, RXC subgroup 

The prepared composite resin blocks were bonded to dentin surfaces without any prior surface preparation with the use of RXC resin cement according to manufacturer’s instructions, followed by light-curing with Demetron A.2 (KERR Corporation, Middle-ton, USA) light-curing unit for 10 seconds.

•Control group, SPC subgroup 

The composite resin blocks were bonded to dentin surfaces without any prior preparation with the use of SPC resin cement according to manufacturer’s instructions, followed by light-curing with the same light-curing unit for 10 seconds as mentioned above.

•Galardin at a high concentration (0.2 mmol), RXC subgroup

Galardin at a concentration of 0.2 mmol was used to prepare the dentin surfaces before bonding of composite resin blocks. Then RXC resin cement was used for boding to dentin surfaces based on manufacturer’s instructions followed by light-curing for 10 seconds using the same light-curing unit.

•Galardin at a high concentration (0.2 mmol), SPC subgroup 

Galardin at a concentration of 0.2 mmol was used to prepare the dentin surfaces before bonding of composite resin blocks. Then SPC resin cement was used for boding to dentin surfaces based on manufacturer’s instructions followed by light-curing for 10 seconds using the same light-curing unit.

•Galardin at a low concentration (5 µmol), RXC subgroup

Galardin at a concentration of 5 μmol was used to prepare the dentin surfaces before bonding of composite resin blocks. Then RXC resin cement was used for boding to dentin surfaces based on manufacturer’s instructions followed by light-curing for 10 seconds using the same light-curing unit.

•Galardin at a low concentration (5 µmol), SPC subgroup

Galardin at a concentration of 5 μmol was used to prepare the dentin surfaces before bonding of composite resin blocks. Then SPC resin cement was used for boding to dentin surfaces based on manufacturer’s instructions followed by light-curing for 10 seconds using the same light-curing unit.

-Shear Bond Strength Testing

Then the samples were stored in distilled water at 37°C for 24 hours and the shear bond strength values were determined in using Universal Testing Machine (Hounsfield Test Equipment, Model H5KS, Surrey, UK) at a strain rate of 0.5 mm/min. Then the bond strength values were converted to MPa using the following formula: Stress (MPa) = N/mm2. The fracture modes were determined under stereomicroscope (Nikon SMZ800, Tokyo, Japan) at ×40, also.

-Statistical Analysis of Data

Normal distribution of data was evaluated with Klomogorov-Smirnov test. Then data were analyzed using two-way ANOVA and a Post-hoc Bonferroni test with SPSS 15 software (α =0.05).

## Results

 Table 2 presents the means of shear bond strength values and standard deviations in the present study.

**Table 2:**
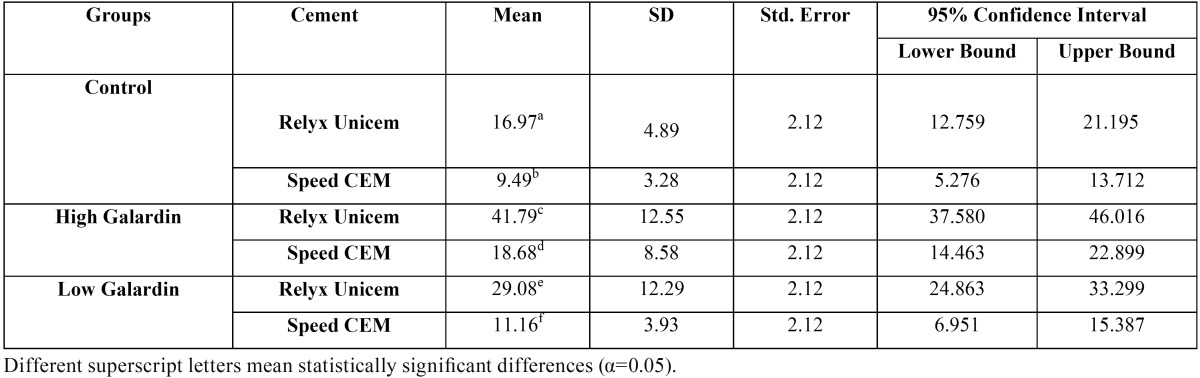
Mean, Standard Deviation (SD) and Descriptive Statistics of Shear bond strength in Study Groups.

Kolmogorov-Smirnov test showed normal distribution of data (*P*>0.05). Two-way ANOVA showed that the effects of different concentrations of galardin (F=32.45, *P*<0.001), cement type (F=87.01. *P*<0.001) and the cumulative effect of different concentrations of galardin and the type of the self-adhesive resin cement on shear bond strength of the samples were significant (F=7.030, *P*=0.001).

Post-hoc Bonferroni test showed that the shear bond strength of galardin at high concentration was significantly higher than that in the control group and galardin at a low concentrations (*P*<0.001). In addition, galardin at a low concentration exhibited higher shear bond strength compared to the control group (*P*=0.005). Furthermore, this test showed a higher shear bond strength value with the use of RXC compared to SPC (*P*<0.001).

Evaluation of the samples under a stereomicroscope showed no traces of the cements on dentin surfaces and all the samples exhibited adhesive failure pattern.

## Discussion

The hypothesis evaluated in the present study was that different concentrations of galardin (0.2 mmol and 5 µmol) were not different in their effect on the immediate bond strength of self-adhesive cements to dentin, which was rejected based on the results of the present study. The results of this study showed that irrigation with galardin at 0.2-mmol and 5-µmol concentrations resulted in a significant increase in shear bond strength of self-adhesive resin cements to dentin and the bond strength increased significantly with an increase in the concentration of the irrigant from 5 µmol to 0.2 mmol.

Alhamdy *et al.* showed that incorporation of some inhibitors of MMPs, including galardin and Batimastat, into the chemical composition of adhesives such as Opti-Bond FL and G-Bond, resulted in a significant increase in the immediate bond strength to dentin. They pointed out that various mechanisms, including assistance in preserving the delicate collagen network and the enzymatic chemical bond between MMPs and their inhibitors or a combination of the two factors above through creation of an adjunctive bonding mechanism, results in an improvement in the micromechanical retention of the adhesive within the collagens network, increasing the bond strength (15).

Galardin and Batimastat have -OH groups (alcoholic groups) in their composition. The protease chain consists of a catalytic Zn, a structural Zn and three calcium ions. The alcoholic group inhibits MMPs by forming a covalent bond between the catalytic Zn ions in the MMP structure and the oxygen atom of the hydroxyl group. The covalent group is one of the strongest inter-molecular bonds and can have a positive effect on the bond strength (19).

Conversely, Breschi *et al.* demonstrated that irrigation of dentin with 2-mmol galardin had no effect on the immediate bond strength and nanoleakage of two-step etch-and-rinse adhesive systems; however, it clearly prevented a decrease in bond strength over time (9). In the present study DMSO (dimethyl sulfoxide) was used to dilute galardin, which is the specific solvent of galardin. DMSO contains methyl groups and sulfur in its chemical structure and is considered a strong solvent for a large number of materials. It has a surface tension similar to that of adhesives and has a low viscosity, is nontoxic, has low vapor pressure and good polarity; and has a capacity to accept hydrogen bonds. Furthermore, Oxygen is very negative in DMSO; therefore, it reacts with cations very well. However, the positive charge is hidden within the molecule. As a result, it cannot solvate the anion through its positive charge. Therefore, the cations do not have the capacity to from a bond and formation of bonds is carried out thorough anions that consist of oxygen and sulfur atoms. It appears the negative ions of DMSO take part in reactions with the positive calcium ions and form a stronger ionic bond (20-23). The primary chemical bonds such as covalent, hydrogen and metallic have a stronger and more durable bonds compared to secondary chemical bonds such as hydrogen and van der Waals bonds due to their higher bond energies. Therefore, this strong bond formed between DMSO anions and tooth Ca+2 can affect the bond strength, increasing it. The fracture mode in all the samples in the present study was adhesive, which might be justified by the surface reaction of these cements with dentin and absence of dentin demineralization and formation of a real hybrid layer by the resin cement on the surface of dentin (24).

Another property of DMSO is its relatively higher surface energy (25). Dillingham *et al.* used DMSO as a criterion to measure the surface energy due to its proper wetting characteristics and low viscosity. They concluded that the proper wetting properties of DMSO are very similar to those of the adhesives and result in an improvement in the performance of adhesive bonds. Therefore, it appears the high surface energy and proper wetting ability of DMSO result in good surface wetting, allowing the cement to form a strong bond with the tooth surface (25).

Another significant finding of the present study was the fact that the shear bond strength of RXC self-adhesive resin cement was significantly higher than that of SPC self-adhesive resin cement. Mazzitelli *et al.* (26) showed that the bond strength of RXC (pH=2.1) was higher than that of G-Cem (pH=2.7). Furthermore, Barcellos *et al.* (27) reported no significant differences between the bond strengths of RXC and Bifix SE with similar pH values. So, it appears the pH value is a factor affecting the bond strength of self-adhesive resin cements. Similarly, Reis *et al.* reported that the penetration depth of the self-etch adhesives into the underlying dentin depends on the pH of the self-etch system. Self-etch achieves with a higher pH value have lower capacity to demineralize the dentin surface compared to self-etch adhesives with lower pH value (28). Ogata *et al.*, too, showed that the structural differences in the dentin prepared with self-etch primers might be related to the pH of self-etch primers (29). In the adhesive systems, the water necessary for the release of hydrogen ions exists in the composition of the adhesive itself. However, in the self-adhesive resin cements, water is provided by the tooth structure. The bulk of self-adhesive cements consist of methacrylate modified by multi-functional phosphoric acid, which eliminate the need for a separate etching step. The acid monomers diffuse through the dentinal tubules, react with the water content of the tissue and are ionized. During penetration of the resin components into dentin, these monomers are neutralized by the calcium in tooth structure and the pH of the material shifts toward a neutral value.

Another factor that might affect the shear bond strength of resin cements is the composition of the monomers of these cements. There are two phosphate groups with a negative charge in the chemical composition of the monomers of RXC self-adhesive resin cement, which affect the positive charges of calcium in the tooth dentin in association with the negative charge of DMSO to form a stronger ionic bond resulting in an increase in shear bond strength. However, in the structure of SPC self-adhesive resin cement, there exists only one phosphate group, which results in a possibly lower synergistic effect with DMSO in comparison with the RC cement, resulting in lower bond strength.

Finally, it can be concluded from the results of the present *in vitro* study that the shear bond strength was higher at a higher concentration of galardin compared to its lower concentration, which was in itself higher than that in the control group. Galardin has an alcoholic group in its composition. Alcohols decrease the diameter of collagen fibers and increase the inter-fibrillar spaces in the hybrid layer, facilitating the penetration of resin monomers. It is suggested that SEM be used for more accurate evaluation of dentin‒resin bond interface. In addition, galardin might affect the quality and quantity of the smear layer, which should be taken into account in future studies.
